# Rapid detection of *Hemophilus influenzae* and *Streptococcus pneumoniae* simultaneously using a duplex recombinase-aided amplification assay directly from invasive clinical samples

**DOI:** 10.3389/fcimb.2025.1631633

**Published:** 2025-11-18

**Authors:** Lin Zhou, Yuyan Xia, Yanling Feng, Bing Du, Xianyi Huang, Wenjian Xu, Jing Li, Fang Fang, Chun Meng, Li Yu, Lijuan Ma, Guanhua Xue, Jing Yuan

**Affiliations:** Capital Center for Children’s Health, Capital Medical University, Capital Institute of Pediatrics, Beijing, China

**Keywords:** recombinase aided amplification (RAA), *Streptococcus pneumoniae*, community-acquired pneumonia (CAP), pediatric infections, point-of-care testing

## Abstract

**Introduction:**

Community-acquired pneumonia (CAP) remains a leading cause of mortality in children under five years of age worldwide. *Streptococcus pneumoniae* and *Haemophilus influenzae* are the most common bacterial pathogens causing CAP that requires hospitalization, highlighting the critical need for a simple, low-cost, and highly sensitive method for rapid diagnosis.

**Methods:**

We developed a duplex recombinase-aided amplification (RAA) assay for the simultaneous detection of *S. pneumoniae* and *H. influenzae*. Following comparative genomic analysis, the conserved lytA gene and omp6 gene were selected as the specific targets for *S. pneumoniae* and *H. influenzae*, respectively. The reaction conditions, including temperature and probe concentration, were optimized.

**Results:**

The established duplex RAA assay can be completed within 10 minutes at a constant temperature of 39°C, with an optimal probe concentration combination of 0.6 μM for *S. pneumoniae* and 0.8 μM for *H. influenzae*. The assay demonstrated high sensitivity, with a limit of detection of 72 copies per reaction for *S. pneumoniae* and 35 copies per reaction for *H. influenzae*.

**Discussion:**

This study presents a rapid and accurate nucleic acid amplification assay for the concurrent detection of two major bacterial pathogens in childhood CAP. The speed, simplicity, and sensitivity of the duplex RAA assay make it a promising tool for early and rapid etiological diagnosis in clinical settings.

## Introduction

Community-acquired pneumonia (CAP) is one of the most common and serious infections in children worldwide, potentially leading to severe complications such as sepsis, acute respiratory distress syndrome, or death (Vaughn et al., 2024). The etiological spectrum of CAP is broad, encompassing a variety of bacteria and viruses (Liu et al., 2023). Among bacterial pathogens, *Haemophilus influenzae* and *Streptococcus pneumoniae* are the two most frequently implicated species. Furthermore, these two pathogens, along with *Neisseria meningitidis*, are leading causes of invasive diseases, including bacteremia, pneumonia, and meningitis. They are also common agents of secondary infections following viral respiratory disease and frequent co-pathogens with *Mycoplasma pneumoniae* (Brueggemann et al., 2021).

*H. influenzae* is a Gram-negative, non-motile bacterium first isolated from the nasopharynx of patients during an influenza epidemic (Wen et al., 2020). *S. pneumoniae* is a Gram-positive, encapsulated diplococcus pathogen first identified from the sputum of a pneumonia patient in 1881 (Zivich et al., 2018). Both organisms are transmitted via respiratory droplets from infected or colonized individuals and subsequently colonize the mucosal surfaces of the nasopharynx and upper airway. This colonization can lead to a range of infections, including otitis media, epiglottitis, sinusitis, pneumonia, meningitis, and bacteremia (Revai et al., 2008). The identification of *S. pneumoniae* and *H. influenzae* is also critical for guiding appropriate antibiotic therapy. A recent study demonstrated that antibiotics were of little benefit for children with acute sinusitis if these bacterial pathogens were absent in the nasopharynx, suggesting that accurate testing for these two bacteria could help reduce unnecessary antibiotic use (Shaikh et al., 2023). Given the clinical significance of these pathogens, the development of accurate and rapid molecular detection methods is crucial for timely diagnosis and treatment.

Despite being the traditional gold standard for diagnosis, bacterial culture often yields a lower positive rate for these two fastidious organisms compared to other pathogens, which is a major constraint in many clinical laboratories (Farajzadeh Sheikh et al., 2021; Sharma et al., 2021). Recombinase-aided amplification (RAA) assay is an isothermal amplification technology known for its high specificity, sensitivity, and portability. The RAA system is composed of three key proteins: a recombinase (which anneals primers to the template DNA), a single-strand DNA binding protein (SSB), and a DNA polymerase (for amplification and extension). The coordinated action of these components with specific primers and fluorescent probes enables a rapid and specific reaction, making the technology highly suitable for point-of-care testing (POCT) and the detection of fastidious bacteria. Recently, RAA has been successfully used to detect various microbial pathogens, such as *Mycoplasma pneumoniae*, *Monkeypox virus*, and *Candida auris* (Xue et al., 2020; Cui et al., 2023; Feng et al., 2024).

In this study, we developed a duplex RAA assay for the simultaneous detection of *S. pneumoniae* and *H. influenzae* in a single tube. We then validated the application of this method using invasive clinical samples and compared its performance against bacterial culture and a previously published real-time PCR method.

## Materials and methods

### Ethics statement and clinical specimen collection

This study was approved by the Ethics Committee of the Capital Institute of Pediatrics. Written informed consent was obtained from the legal guardian of each pediatric patient prior to sample collection.

A total of 168 specimens were collected from pediatric patients at the Capital Center for Children’s Health, Capital Medical University, Capital Institute of Pediatrics, Beijing, China, between January 2023 and December 2024. Each specimen was obtained from a unique patient, and the collection comprised 160 bronchoalveolar lavage fluid (BALF) samples and 8 cerebrospinal fluid (CSF) samples.

### Bacterial and viral strains/DNA

Standard reference strains of *H. influenzae* (non-encapsulated, ATCC 49247; and encapsulated, ATCC 9334) and *S. pneumoniae* (ATCC 49619) were used to establish and validate the assay. To evaluate analytical specificity, a panel of nucleic acids from common respiratory pathogens stored at the Capital Institute of Pediatrics was tested. This panel included: *Mycoplasma pneumoniae, Staphylococcus aureus, Klebsiella pneumoniae, Pseudomonas aeruginosa, Escherichia coli, Legionella pneumophila, Listeria monocytogenes, Acinetobacter baumannii, Mycobacterium tuberculosis, Bordetella pertussis, Streptococcus mitis, Streptococcus oralis, Streptococcus agalactiae, Streptococcus mutans, Streptococcus parasanguinis, Streptococcus sanguinis, Streptococcus salivarius*, *influenza* A and B viruses, *parainfluenza viruses*, *adenoviruses*, *respiratory syncytial virus*, *human metapneumovirus*, *human bocavirus*, and *rhinovirus* (**Table 1**).

**Table 1:** Bacterial or virus strain/DNA types used in this study.

Strain/DNA	Source
*Streptococcus pneumoniae* ATCC49619	Our microorganism center
*Haemophilus influenzae* ATCC49247	Our microorganism center
*Haemophilus influenzae* ATCC9334	Our microorganism center
Influenza A	Our microorganism center
Influenza B	Our microorganism center
Parainfluenza viruses (PIV)	Our microorganism center
Adenoviruses (ADV) DNA	Clinical isolate DNA
Respiratory syncytial virus (RSV)	Our microorganism center
Human metapneumovirus (HMPV)	Our microorganism center
Human bocavirus (BoV)	Our microorganism center
Rhinovirus (Rh)	Clinical isolate DNA
*Mycoplasma pneumoniae* M129	Our microorganism center
*Mycoplasma pneumoniae* FH	Our microorganism center
*Legionella pneumophila* ATCC33823	Our microorganism center
*Listeria monocytogenes*	Clinical isolate DNA
*Staphylococcus aureus* ATCC29213	Our microorganism center
*Klebsiella pneumoniae* ATCC BAA-2146	Our microorganism center
*Pseudomonas aeruginosa*	Our microorganism center
*Escherichia coli* ATCC 25922	Our microorganism center
*Acinetobacter baumannii*	Our microorganism center
*Mycobacterium tuberculosis*	Clinical isolate DNA
*Bordetella pertussis*	Clinical isolate DNA
*Streptococcus mitis*	Clinical isolate DNA
*Streptococcus agalactiae*	Clinical isolate DNA
*Streptococcus salivarius*	Clinical isolate DNA

### DNA extraction

Total genomic DNA was extracted from bacterial cultures and clinical specimens using the QIAamp DNA Mini Kit (Qiagen, Hilden, Germany) according to the manufacturer’s instructions. The extracted DNA was eluted in 150 μL of nuclease-free water and stored at −80°C until further use. DNA concentrations were measured using a NanoDrop spectrophotometer (Thermo Fisher Scientific, USA). DNA copy numbers were calculated using the following formula: DNA copy number (copies/μL) = [6.02 × 10^23^ × plasmid concentration (ng/μL) × 10^−9^]/[DNA length (in nucleotides) × 660].

### Primer and probe design

Conserved regions within the *lytA* gene of *S. pneumoniae* and the *omp6* gene of *H. influenzae* were selected as amplification targets. Specific primers and probes were designed following the principles of the recombinase-aided amplification (RAA) assay. The specificity of all primers and probes was confirmed in silico using NCBI’s Primer-BLAST tool. Potential secondary structures, such as primer-dimers and hairpins, were analyzed using the online OligoEvaluator software. All oligonucleotides were synthesized and purified via high-performance liquid chromatography by Sangon Biotech (Shanghai, China).

### Recombinant plasmid construction

The full-length sequences of the *lytA* and *omp6* genes were PCR-amplified and subsequently cloned into the pUC57 vector (Tiangen Biotech Co., Ltd., Beijing, China). These recombinant plasmids served as standards. Tenfold serial dilutions were prepared to create standards with concentrations ranging from 10^7^ to 10^0^ copies/μL, which were then stored at −80°C.

### Duplex RAA assay procedure

RAA assays were conducted in a total reaction volume of 50 μL using a commercial RAA kit (Jiangsu Qitian Bio-Tech Co., Ltd., China). Each reaction mixture contained 2 µL of template DNA, 25 µL of reaction buffer, 15.7 µL of DNase-free water, 2.5 µL of 280 mM magnesium acetate, 2.1 µL of each *lytA* primer (10 µM), 2.1 µL of each *omp6* primer (10 µM), and a combination of *lytA* (VIC-labeled) and *omp6* (FAM-labeled) probes. Probe concentrations were optimized by testing various combinations (0.6/0.8 µM, 0.6/0.6 µM, and 0.8/0.6 µM). The mixture was added to a tube containing the lyophilized RAA enzyme pellet. The tubes were vortexed and centrifuged, then incubated in a B6100 Oscillation Mixer for 4 min. Finally, the tubes were transferred to a real-time fluorescence detector (QT-RAA-1620) for signal acquisition at 39°C for 20 min.

### Analysis of sensitivity and specificity

The analytical sensitivity of the RAA assay was determined using tenfold serial dilutions of the recombinant plasmid ranging from 10^7^ to 10^0^ copies/μL. The analytical specificity was evaluated by testing the RAA assay against the panel of non-target microbial pathogens listed in **Table 1**. Standard *S. pneumoniae* and *H. influenzae* DNA were used as positive controls, and nuclease-free water served as the negative control.

### Clinical specimen evaluation

All 168 clinical specimens were tested in parallel using the newly developed duplex RAA assay, the previously published real-time PCR assays for *S. pneumoniae* (Carvalho et al., 2007) and *H. influenzae* (Abdeldaim et al., 2010), and conventional bacterial culture. The primers and probes used for real-time PCR are listed in **Table 2**. PCR cycling conditions were performed as described in the respective publications (Carvalho et al., 2007; Abdeldaim et al., 2010).

**Table 2:** Primers and probes used in this study.

Name	Sequence (5’-3’)	Source
LytA-F-RAA	TCAAAGTAGTACCAAGTGCCATTGATTTTC	
LytA-R-RRA	AGCCGTGAGCAGTTTAAGCYATGATATTGAGAA	
LytA-P-RAA	TAAGAGCCGTCTGARTGTACGTACCAGTAGCCAG[VIC-dT][THF][BHQ-dT] CATTCTTCTGCCAGCCT	
Omp6-F-RAA	CAATGGTGCTGCTCAAACTTTTGGCGGATAC	
Omp6-R-RAA	CTTCTACTAATACTTTAGCAGCTGGCGTTGCA	
Omp6-P-RAA	CTGATCTTCAACAACGTTACAACACCGTATAT[FAM-dT][THF][BHQ-dT] GGTTTTGATAAATAC	
LytA-F-qPCR	5′-ACGCAATCTAGCAGATGAAGCA-3′	Ref (Carvalho et al., 2007)
LytA-R-qPCR	5′-TCGTGCGTTTTAATTCCAGCT-3′	
LytA-P-qPCR	5′-FAM-GCCGAAAACGCTTGATACAGGGAG-3′-BHQ1	
Omp6-F-qPCR	5’-CCAGCTGCTAAAGTATTAGTAGAA G-3’	Ref (Abdeldaim et al., 2010)
Omp6-R-qPCR	5’-TTCACCGTAAGATACTGTGCC-3’	
Omp6-P-qPCR	5’-VIC-CAGATGCAGTTGAAGGTTATTTAG-3′-BHQ1	

### Statistical analysis

Statistical analyses were performed using SPSS version 21.0. The diagnostic performance of the duplex RAA assay, including its sensitivity, specificity, positive predictive value (PPV), and negative predictive value (NPV), was evaluated by the chi-square test using real-time PCR as the gold standard. Based on the confusion matrix, samples were classified as true positive (TP), false positive (FP), true negative (TN), or false negative (FN). The detailed metrics were calculated as follows: Sensitivity = TP/(TP + FN); Specificity = TN/(TN + FP); PPV = TP/(TP + FP); NPV = TN/(TN + FN).

## Results

### Establishment and workflow of the duplex RAA method

**Figure 1:**
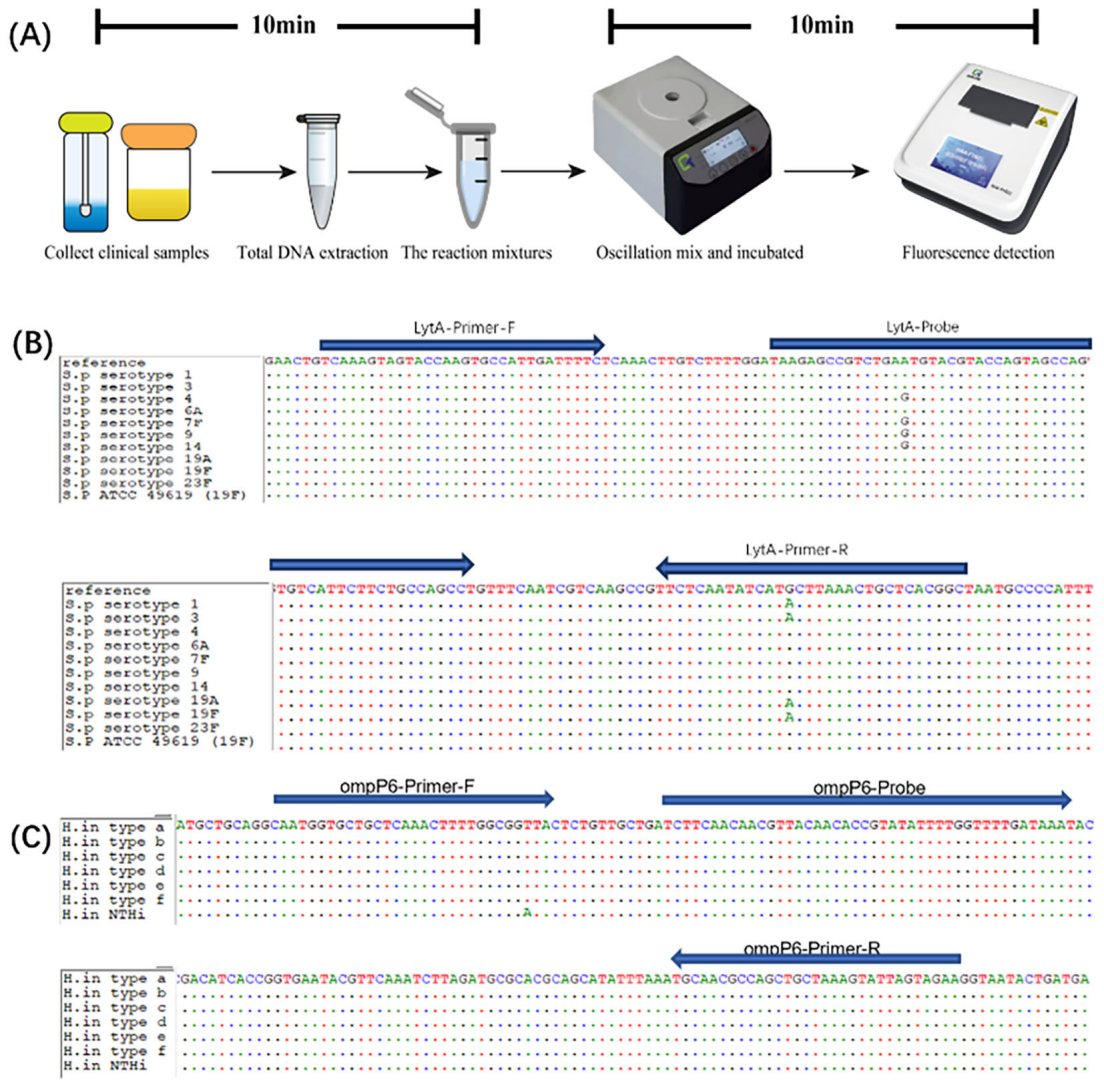
Establishment of the duplex recombinase-aided amplification (RAA) method. **(A)** Workflow of the duplex RAA method. **(B)** Alignment of the *lytA* gene among different *S. pneumoniae* strains, including serotype 1, type 3, type 4, type 6A, type 7F, type 9C, type 14, type 19A, type 19F, and type 23F. **(C)** Alignment of the omp6 gene among different *H influenzae* strains, including typeable (encapsulated a–f) and nontypeable (nonencapsulated) strains.

The workflow for the duplex recombinase-aided amplification (RAA) assay was streamlined for rapid sample-to-result analysis. Following DNA extraction from clinical specimens, the prepared reaction mixture—containing primers and probes for both *S. pneumoniae* (VIC channel) and *H. influenzae* (FAM channel)—was incubated in an isothermal instrument. Positive results were typically observed within 10 min, with a total turnaround time of under 20 min (**Figure 1A**).

To ensure comprehensive detection across diverse strains—including typeable (a-f) and non-typeable *H. influenzae* (Bakaletz and Novotny, 2018) and the numerous serogroups of *S. pneumoniae* (Davies et al., 2022)—we selected highly conserved target regions. Through comparative genomic analysis, the *omp6* gene of *H. influenzae* and the *lytA* gene of *S. pneumoniae* were chosen. Specific primers and probes were designed within these conserved regions, with certain single-nucleotide polymorphism (SNP) loci among different types accounted for by introducing degenerate bases (**Figures 1B, C**). The potential for primer-dimer and hairpin formation was assessed using online tools from Integrated DNA Technologies (IDT), and no significant secondary structures were predicted.

### Optimization of the duplex RAA reaction system

**Figure 2:**
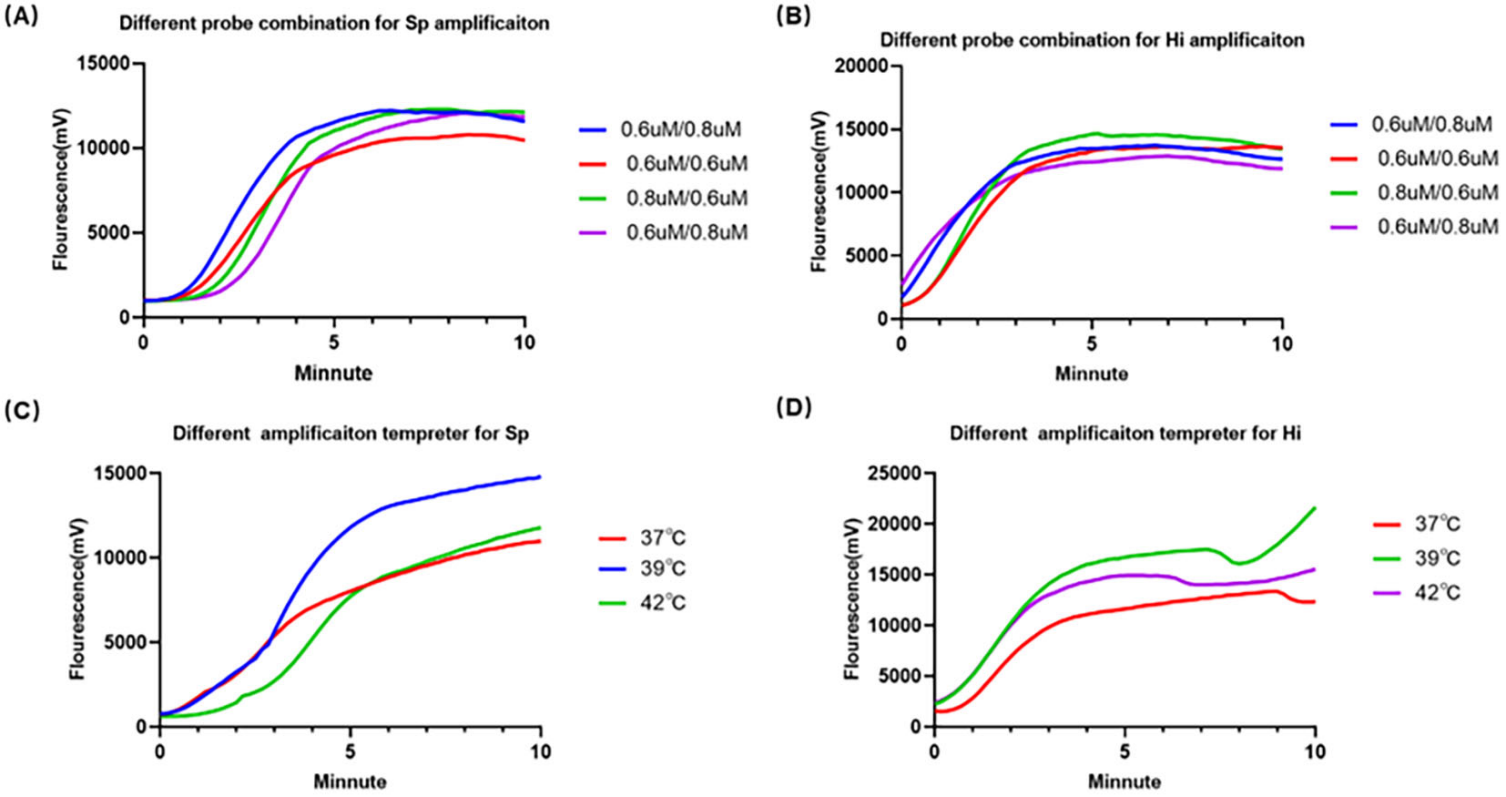
Optimization of the duplex RAA assay parameters. **(A)** Relative fluorescence curves for different RAA probe combinations for *S. pneumoniae*. **(B)** Relative fluorescence curves for different RAA probe combinations for *H influenzae*. **(C)** Relative fluorescence curves at different incubation temperatures (37°C, 39 °C,42°C for *S. pneumoniae*. **(D)** Relative fluorescence curves at different incubation temperatures (37°C, 39 °C,42°C for *H influenzae*.

To achieve optimal amplification efficiency, we systematically tested different probe concentrations and reaction temperatures. Various combinations of the *lytA* (*S. pneumoniae*) and *omp6* (*H. influenzae*) probes were evaluated. A final concentration of 0.8 μM for the *lytA* probe and 0.6 μM for the *omp6* probe yielded the most robust and consistent amplification signals. Subsequently, this optimal probe combination was tested at 37°C, 39°C, and 42°C. The reaction performed at 39°C produced the highest fluorescence intensity (**Figure 2**). Consequently, these optimal conditions (0.8/0.6 μM probes at 39°C) were used for all subsequent experiments.

### Analytical specificity and sensitivity

**Figure 3:**
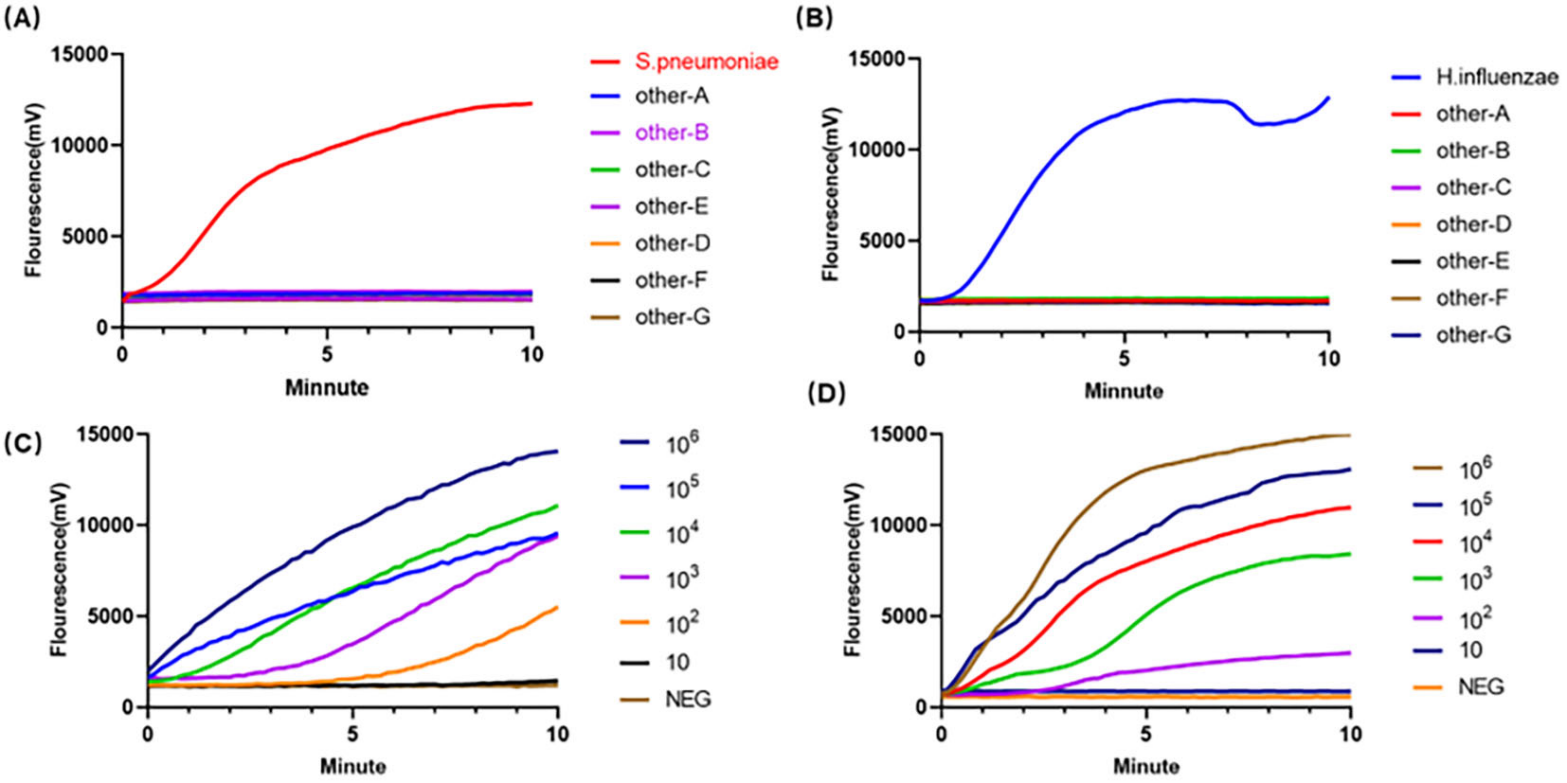
Specificity and sensitivity of the duplex RAA assay for *S. pneumoniae* and *H influenzae* detection. **(A)** Specificity of the RAA assay for *S. pneumoniae*. **(B)** Specificity of the RAA assay for *H influenzae*. **(C)** Sensitivity of the RAA assay for *S. pneumoniae*. **(D)** Sensitivity of the RAA assay for *H influenzae*.

The analytical specificity of the duplex RAA assay was confirmed using a panel of common respiratory pathogens. As shown in **Figure 3**, amplification signals were generated exclusively from *S. pneumoniae* and *H. influenzae* templates. No cross-reactivity was observed with any of the other bacterial or viral nucleic acids tested, nor in the no-template (water) control, demonstrating 100% specificity.

The analytical sensitivity was determined using tenfold serial dilutions of recombinant plasmids containing the target genes. A clear fluorescence signal was detected across dilutions ranging from 1 × 10^7^ down to 1 × 10¹ copies/reaction. The limit of detection (LOD) was established as 72 copies/reaction for *S. pneumoniae* and 35 copies/reaction for *H. influenzae*.

### Evaluation of the duplex RAA assay on clinical samples

The clinical performance of the duplex RAA assay was evaluated using 168 invasive specimens, with results compared against those from a validated real-time PCR assay and conventional bacterial culture. The RAA assay identified 18 specimens as positive for *S. pneumoniae* and 21 for *H. influenzae*. Among these, six specimens were positive for both pathogens. When real-time PCR was used as the diagnostic gold standard, the duplex RAA assay for the two pathogens showed 100% positive predictive value (PPV) and negative predictive value (NPV). In contrast, bacterial culture yielded significantly fewer positive results, detecting only nine cases of *S. pneumoniae* and seven cases of *H. influenzae* (**Table 3**).

**Table 3:** Comparison of RAA assay, real-time PCR and culture for *S.pneumoniae* and *H. influenzae* detection of clinical samples.

Method	*S.pneumoniae* positive/ (%)	*S.pneumoniae* negative/ (%)	*H.influenzae* positive/ (%)	*H.influenzae* negative/ (%)	Total
RAA assay	18 (10.71%)	150 (89.29%)	21 (12.5%)	147 (87.5%)	168
Real-time PCR	18 (10.71%)	150 (89.29%)	21 (12.5%)	147 (87.5%)	168
Culture	9 (5.36%)	159 (94.64%)	7 (4.17%)	161 (95.83%)	168

## Discussion

*Streptococcus pneumoniae* and *Haemophilus influenzae* are common inhabitants of the human nasopharyngeal microflora but can migrate to sterile sites to cause invasive disease (Eichner et al., 2021). Recent metagenomic studies of BALF from children have identified *S. pneumoniae* and *H. influenzae* as dominant pathogens (Yang et al., 2022). Similarly, advanced sequencing of cerebrospinal fluid in resource-limited settings has shown *S. pneumoniae* to be a primary cause of bacterial meningitis (Pallerla et al., 2022). Given the clinical importance of these bacteria, a rapid and accurate detection method applicable directly to clinical samples is essential for early diagnosis. Any diagnostic delay can increase mortality, prolong hospital stays, and elevate healthcare expenditures (Pallerla et al., 2022).

Since both pathogens can asymptomatically colonize the nasopharynx, a positive result from noninvasive samples (e.g., pharyngeal swabs) can make it difficult to distinguish between pathogenic infection and carriage. To address this diagnostic ambiguity, our study focused on developing a duplex recombinase-aided amplification (RAA) assay and evaluating it on invasive clinical samples (BALF and CSF). This approach provides a more conclusive diagnostic basis for clinicians. The RAA assay achieves amplification in approximately 10 min, significantly reducing the turnaround time compared with real-time PCR and enhancing its utility in clinical settings.

For developing robust diagnostic methods, selecting highly conserved target sequences is crucial to ensure comprehensive detection across numerous serotypes. Given the genomic variability among different genotypes and serotypes of these pathogens, this initial step is critical for accuracy. Previous research on *S. pneumoniae* has utilized various gene targets, including those encoding pneumolysin (*ply*), autolysin (*lytA*), Spn9802, capsular polysaccharide biosynthesis proteins (*cps*), and pneumococcal surface antigen A (*psaA*) (Blaschke, 2011; Wessels et al., 2012; Chang et al., 2021). Notably, the *lytA* gene, which encodes a cell wall hydrolase, is a recognized virulence factor due to its role in releasing highly inflammatory cell wall components and pneumolysin. Consequently, it has been repeatedly validated as an effective and reliable detection target (Seki et al., 2005). For *H. influenzae*, specific genes such as the outer membrane protein P6 gene (*omp6*) and 16S rRNA have been successfully targeted (Maleki et al., 2020; Wang et al., 2022). For instance, Cao et al. established an isothermal amplification technology based on the *omp6* gene, achieving sensitive detection by coupling multiple cross displacement amplification with a nanoparticle-based lateral flow biosensor (Cao et al., 2021).

Informed by these previous studies, we selected the *lytA* and *omp6* genes as target markers for our assay. Sequence alignments confirmed that these genes exhibit high sequence homology across diverse serotypes. Consequently, primers and probes were designed based on these conserved regions. The resulting single-tube duplex assay demonstrated high specificity for various strains of *H. influenzae* and *S. pneumoniae*, with no cross-reactivity observed against other common respiratory pathogens. The analytical sensitivity was determined to be 72 copies per reaction for *S. pneumoniae* (*lytA*) and 35 copies per reaction for *H. influenzae* (*omp6*). This performance is comparable to the sensitivity of previously reported real-time PCR assays, which typically ranges from 10 to 100 copies per reaction (Abdeldaim et al., 2009; Meyler et al., 2012; Hajia et al., 2014), indicating that our RAA assay exhibits competitive detection sensitivity.

To evaluate the clinical utility of this method, we analyzed 168 invasive clinical samples. The results from both the RAA and real-time PCR assays demonstrated 100% concordance, with positive detection rates of 10.71% for *S. pneumoniae* and 12.5% for *H. influenzae*. In contrast, the positive rates for bacterial culture were only 5.36% and 4.17%, respectively. Notably, for samples that were culture-negative but RAA-positive, the amplification dynamics suggested a low bacterial load, likely below the detection threshold of conventional culture. This finding highlights the superior sensitivity of the RAA assay. Furthermore, we observed a high rate of co-infection: 33.3% (6/18) of the *S. pneumoniae*-positive samples were also co-infected with *H. influenzae*. As previous studies have indicated, co-infection with these two pathogens can exacerbate pneumonia and increase mortality in pediatric patients (Pneumonia Etiology Research for Child Health (PERCH) Study Group, 2019). These findings underscore the clinical importance of simultaneous detection.

However, this study has limitations. The primary limitation is the qualitative nature of the RAA assay. Although the amplification curve can provide a semi-quantitative indication of bacterial load, it does not offer the precise quantification achievable with methods such as real-time PCR.

In summary, we have developed a duplex RAA assay for the simultaneous detection of *H. influenzae* and *S. pneumoniae* in clinical samples, demonstrating high specificity and sensitivity. This assay offers significant advantages, including simplified reaction conditions, a reduced turnaround time, and lower cost compared with traditional culture methods and real-time PCR. Therefore, it holds substantial potential for clinical application, particularly for the rapid analysis of invasive specimens.

## Data Availability

Publicly available datasets were analyzed in this study. This data can be found here: https://www.ncbi.nlm.nih.gov/.
