# Correction: Rapid detection of *Hemophilus influenzae* and *Streptococcus pneumoniae* simultaneously using a duplex recombinase-aided amplification assay directly from invasive clinical samples

**DOI:** 10.3389/fcimb.2025.1749183

**Published:** 2025-12-08

**Authors:** Lin Zhou, Yuyan Xia, Yanling Feng, Bing Du, Xianyi Huang, Wenjian Xu, Jing Li, Fang Fang, Chun Meng, Li Yu, Lijuan Ma, Guanhua Xue, Jing Yuan

**Affiliations:** Capital Center for Children’s Health, Capital Medical University, Capital Institute of Pediatrics, Beijing, China

**Keywords:** recombinase aided amplification (RAA), *Streptococcus pneumoniae*, community-acquired pneumonia (CAP), pediatric infections, point-of-care testing

Author “Wenjian Xu” was erroneously assigned as equal contributing author. The original version of this article has been updated.

Author “Lin Zhou” was erroneously omitted as equal contributing author.

The original version of this article has been updated.

